# Effect of Europium Addition on the Microstructure and Dielectric Properties of CCTO Ceramic Prepared Using Conventional and Microwave Sintering

**DOI:** 10.3390/molecules28041649

**Published:** 2023-02-08

**Authors:** Gecil Evangeline T, Raja Annamalai A, Pavel Ctibor

**Affiliations:** 1 School of Mechanical Engineering, Vellore Institute of Technology, Vellore 632014, India; 2 Centre for Innovative Manufacturing Research, Vellore Institute of Technology, Vellore 632014, India; 3 The Czech Academy of Sciences, Institute of Plasma Physics, U Slovanky 1a, 182 00 Prague, Czech Republic

**Keywords:** europium, CCTO, dielectric loss, microstructure, conventional sintering, microwave sintering

## Abstract

In this work, Eu_2_O_3_-doped (CaCu_3_Ti_4_O_12_)_x_ of low dielectric loss have been fabricated using both conventional (CS) and microwave sintering (MWS), where x = Eu_2_O_3_ = 0.1, 0.2, and 0.3, respectively. According to X-ray diffraction (XRD) and scanning electron microscope (SEM) reports, increasing the concentration of Eu^3+^ in the CCTO lattice causes the grain size of the MWS samples to increase and vice versa for CS. The X-ray photoelectron spectroscopy (XPS) delineated the binding energies and charge states of the Cu^2+^/Cu^+^ and Ti^4+^/Ti^3+^ transition ions. Energy dispersive spectroscopy (EDS) analysis revealed no Cu-rich phase along the grain boundaries that directly impacts the dielectric properties. The dielectric characteristics, which include dielectric constant (ε) and the loss (tan δ), were examined using broadband dielectric spectrometer (BDS) from 10 to 10^7^ Hz at ambient temperature. The dielectric constant was >10^4^ and >10^2^ for CS and MWS samples at x > 0.1, respectively, with the low loss being constant even at high frequencies due to the effective suppression of tan δ by Eu^3+^. This ceramic of low dielectric loss has potential for commercial applications at comparatively high frequencies.

## 1. Introduction

The electrical features of calcium copper titanium oxide (CaCu_3_Ti_4_O_12_/CCTO), such as an enhanced breakdown field [1,2] and nonlinear current-voltage characteristics [3,4,5], have piqued the interest of the research community. The CCTO’s perovskite cubic structure, in which the TiO_6_ is octahedrally arranged, along with the copper’s square planar coordination, have been its most distinctive aspect [6,7]. Moreover, they have been investigated for use as photocatalysts in effluent water treatment [8], humidity sensors [9], photoelectrochemical cells (PECs) [10], and energy storage devices [11]. The inherent internal barrier layer capacitance (IBLC) effect and one-step simplicity of CCTO production have made it a popular choice among dielectric materials [12,13].

Many intrinsic and extrinsic characteristics, including microstructure, processing time, cooling cycle, redox reactions, sintering temperature, and heating rate, have influenced the IBLC model [14,15,16]. Although CCTO offers intriguing qualities, there are numerous limitations in terms of choosing the appropriate high-quality and pure precursors by means of environmentally friendly synthesis methods, shortening the processing time, and changing the particles’ composition. To improve cost efficiency and shorten processing times for high-quality ceramic powders, new and improved methods of synthesis have been presented as an alternative to conventional methods [17,18]. It has been made possible by recent technological advancements to produce a high-quality end product using pressure and unconventional heating methods. The heating process has been upgraded using sintering techniques, which combine pressure with electric discharge and other sources, such as microwave irradiation. However, the semiconducting grains and the insulating grain boundaries change over time due to processing inputs and the presence of other elements; therefore, all of the above-mentioned parameters play a significant role [19,20,21].

The characteristics of the ceramic may alter depending on the sintering parameters due to the segregation of the Cu-rich phase promoted by the reduction reaction of Ti^4+^ and Cu^2+^ at high sintering temperature [22,23]. Substitution with rare earth elements, such as lanthanum and europium, has proven to be an effective technique to increase the dielectric permittivity of CCTO, while maintaining a steady reduction in the dielectric losses at high frequencies due to the ion size effect. As their radius is greater than the inclusion site, rare earth elements have a distinct advantage. Their enhanced dielectric characteristics and reduced polarization-induced dielectric loss offer to be a promising candidate in microelectronic device production [24,25,26]. In this study, europium-doped CCTO ceramics have been prepped with three distinct compositions (0.1, 0.2, and 0.3) using both traditional and state-of-the-art techniques, such as microwave sintering. Multiple characterization techniques, including scanning electron microscopy (SEM), X-ray diffractometer (XRD), energy dispersive spectroscopy (EDS), X-ray photoelectron spectroscopy (XPS), and broadband dielectric spectrometer (BDS), were used to examine europium’s impact on CCTO’s microstructure and dielectric characteristics. To further comprehend Eu^3+^ in lowering the dielectric loss at high frequencies, this research will be an outset.

## 2. Results

### 2.1. XRD Analysis

**Figure 1:**
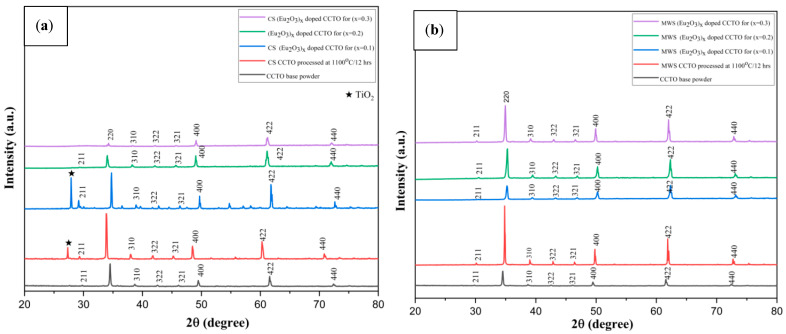
X-ray diffraction pattern of Eu-doped CCTO samples at 1100 °C. (**a**) Conventional sintered for 12 h, (**b**) microwave sintered for 30 min.

All of the europium-doped samples showed CCTO-related peaks in the X-ray diffraction pattern (shown in Figure 1a) using the JCPDS file number 01-070-5808. However, at x = 0.1, a faint hint of secondary peaks becomes visible. It has been hypothesized that the varying ionic radii of the elements were responsible for this modest shift in Bragg’s peak to a higher angle—Eu^3+^ (94.7 pm) on the CCTO lattice. Ca^2+^ voids in Calcium Copper Titanate have been filled by Eu^3+^, which can be perceived from the XRD data. Microwave sintered samples (shown in Figure 1b) displayed a pure single cubic phase of CCTO, with peaks matching those of pure CCTO and without evidence of impurities or secondary phases.

### 2.2. Microstructure Analysis Using SEM and EDS

**Figure 2:**
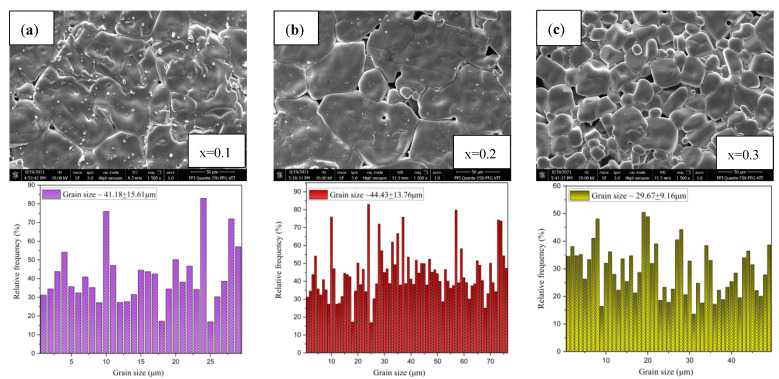
SEM images, along with the histograms of grain size distribution, of conventional sintered europium-doped CCTO at (**a**) x = 0.1, (**b**) x = 0.2, and (**c**) x = 0.3 for 12 h at 1100 °C.

**Figure 3:**
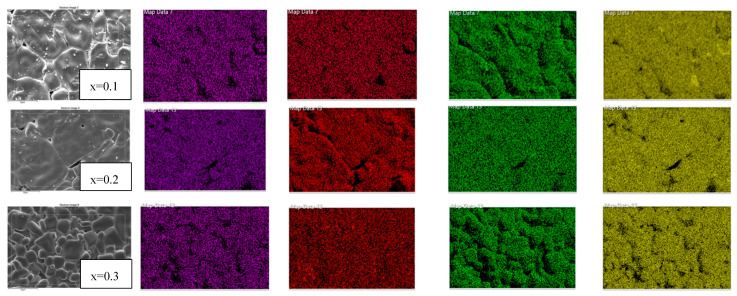
Energy dispersive spectroscopy (EDS) mapping of europium-doped samples CS at x = 0.1, 0.2, and 0.3, respectively.

**Figure 4:**
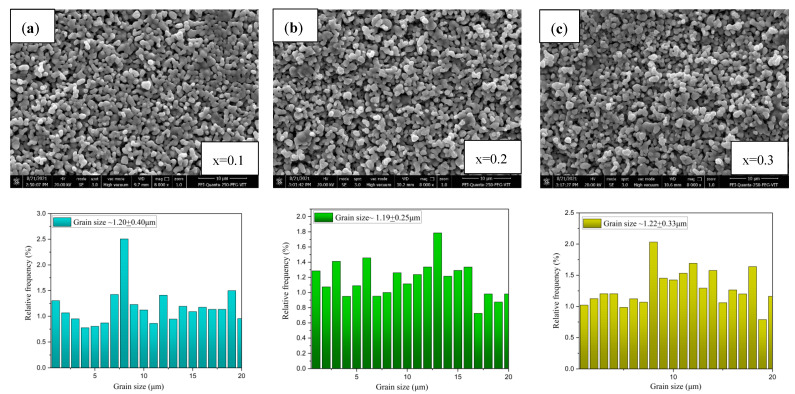
SEM images, along with the histograms of grain size distribution, of microwave sintered europium-doped CCTO at (**a**) x = 0.1, (**b**) x = 0.2, and (**c**) x = 0.3 for 30 min at 1100 °C.

**Figure 5:**
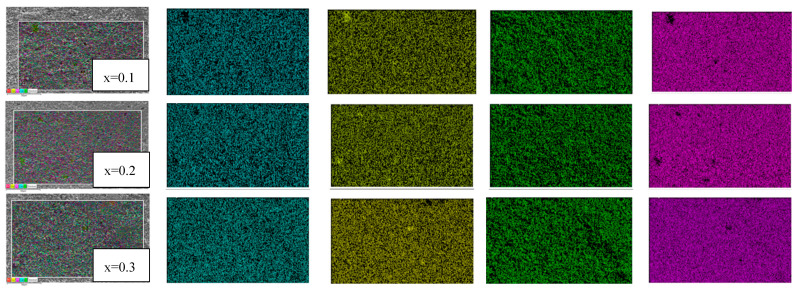
Energy dispersive spectroscopy (EDS) mapping of europium-doped samples MWS at x = 0.1, 0.2, and 0.3, respectively.

Scanning electron microscope images, shown in Figure 2, Figure 3, Figure 4 and Figure 5 were used to examine the microstructure. The testing revealed that grains of the doped samples were usually larger than the pure CCTO samples. Image J software was used to determine the maximum average grain size of CS and MWS europium-doped samples, and the reported value was verified using the linear intercept approach. It can be observed in Figure 2b that at x = 0.2, the average grain size was 44.43 ± 13.76 μm, indicating that the grains were quite sizable. Similarly, Figure 2a–c has illustrated grain sizes of 41.18 ± 15.61 μm and 29.67 ± 9.16 μm at x = 0.1 and 0.3 with minimum grain size at x = 0.3 due to the high Eu content. The solute drag mechanism promoted by the rare earth cations [27,28,29] may account for the smaller grains seen in CS samples at x = 0.3. Grain size and structure evolution have also been affected by Eu^3+^ occupancy in the CCTO structure [30]. The average grain size of microwave sintered (MWS) samples revealed an increase in europium content between x = 0.1 (~1.20 ± 0.40 μm) and x = 0.3 (~1.22 ± 0.33 μm). Although the grain size enlarged as the level of Eu content increased, there has been negligible reduction in grain size (~1.19 ± 0.25 μm) at x = 0.2. From the SEM images, it can be inferred that the grain size decreases with an increase in the Eu content. On the other hand, as shown in Figure 4a–c, the grain size of MWS samples increased with the increase in Eu dopant content, which has been accounted for microwave heating. Figure 3 and Figure 5 depict the energy dispersive spectroscopy (EDS) analysis of the elemental distribution in CS and MWS europium-doped CCTO samples. Elements, such as Ca, Ti, and O, have all been found to be uniformly distributed across the grain region, and the Cu-rich phase does not segregate along the grain boundaries.

### 2.3. X-ray Photoelectron Spectroscopy (XPS) Analysis

**Figure 6:**
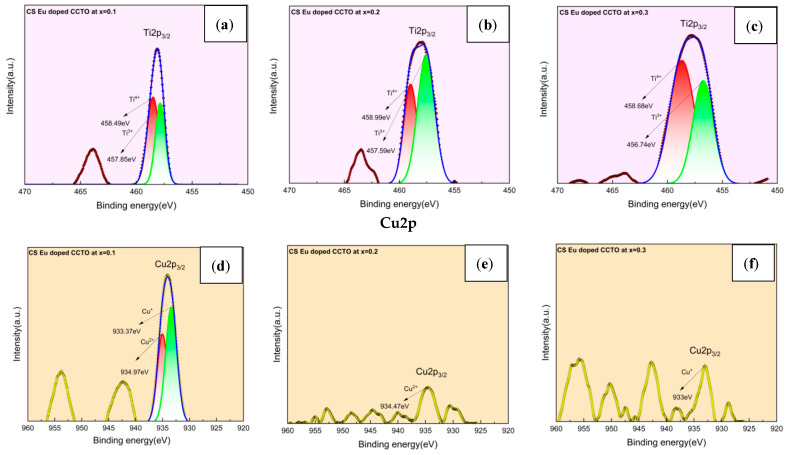
(**a**–**c**) Ti2p and (**d**–**f**) Cu2p spectra of CS europium-doped CCTO samples from x = 0.1 to 0.3.

**Figure 7:**
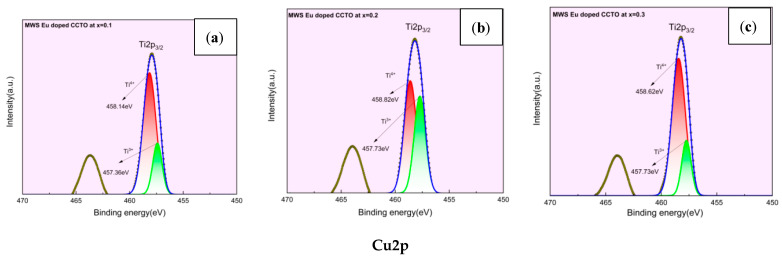

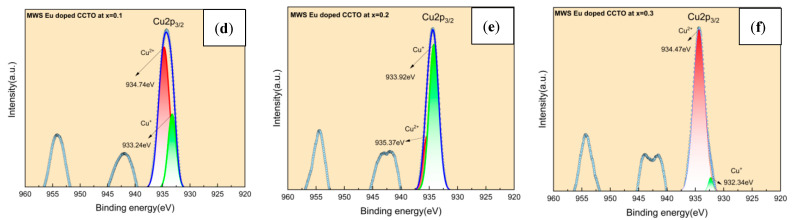
(**a**–**c**) Ti2p and (**d**–**f**) Cu2p spectra of MWS europium-doped CCTO samples from x = 0.1 to 0.3.

The binding energies and charge state of the Cu^2+^/Cu^+^ and Ti^4+^/Ti^3+^ transition ions were estimated through Gaussian–Lorentzian peak fitting software program and illustrated in Figure 6a–f and Figure 7a–f. Binding energies (BE) of Ti^4+^/Ti^3+^ for CS samples have been 458.49/457.85 eV, 458.99/457.59 eV, and 458.68/456.74 eV, respectively. While, at = 0.1, the BE of Cu^2+^/Cu^+^ has been 934.97 and 933.37 eV. Accordingly, the binding energies for Cu^2+^ and Cu^+^ were 934.47/933.00 eV at x = 0.2 and 0.3. As in the case of MWS samples, the Ti and Cu spectra displayed BE of 458.14/457.36 eV, 458.82/457.73 eV, 458.62/457.62 eV for Ti^4 +^ /Ti^3+^ and 934.74/933.24 eV, 935.37/933.92 eV, 934.47/932.34 eV for Cu^2+^/Cu^+^ from x = 0.1 to 0.3. Oxygen loss during sintering at 1100 °C and the charge compensation process generated by oxygen vacancies have been computed for the difference in the binding energies. It has been evidenced that the unbound oxygen, formed as a result of charge compensation mechanism of Cu and Ti, fosters semiconductivity of the grains. These semiconducting grains surrounded by insulating grain boundaries impart high permittivity to the material. The presence of Cu^+^ and Ti^3+^ from reduction processes also initiates the conductivity inside the grains, leading to the IBLC effect, which in turn improves the dielectric characteristics [31,32,33,34,35].

### 2.4. Dielectric Characteristics

**Figure 8:**
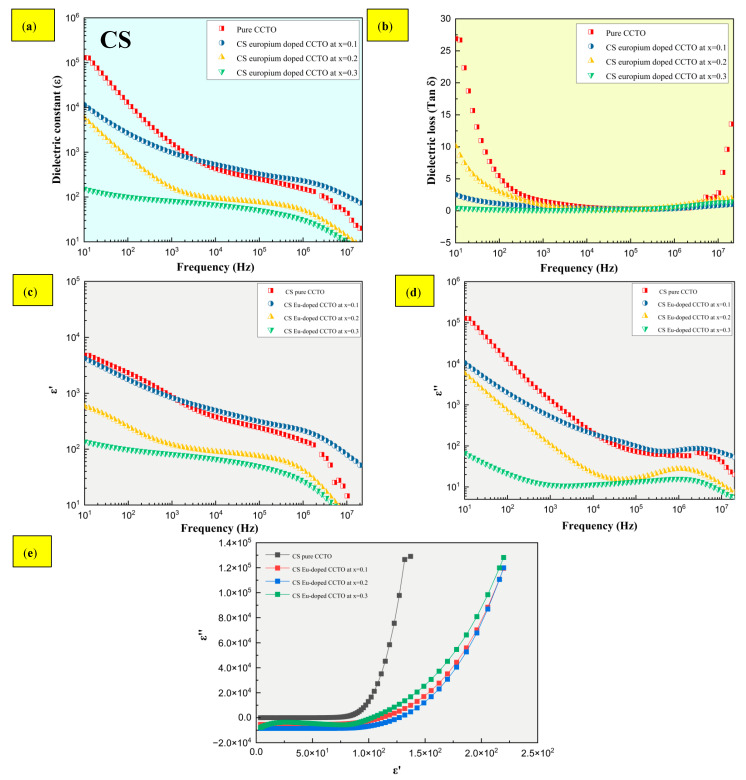
Dielectric properties of CS europium-doped samples from x = 0.1 to 0.3 measured at room temperature. (**a**) ε/Frequency, (**b**) tan δ/frequency, (**c**,**d**) dielectric relaxation peaks, and (**e**) Cole-Cole plot.

**Figure 9:**
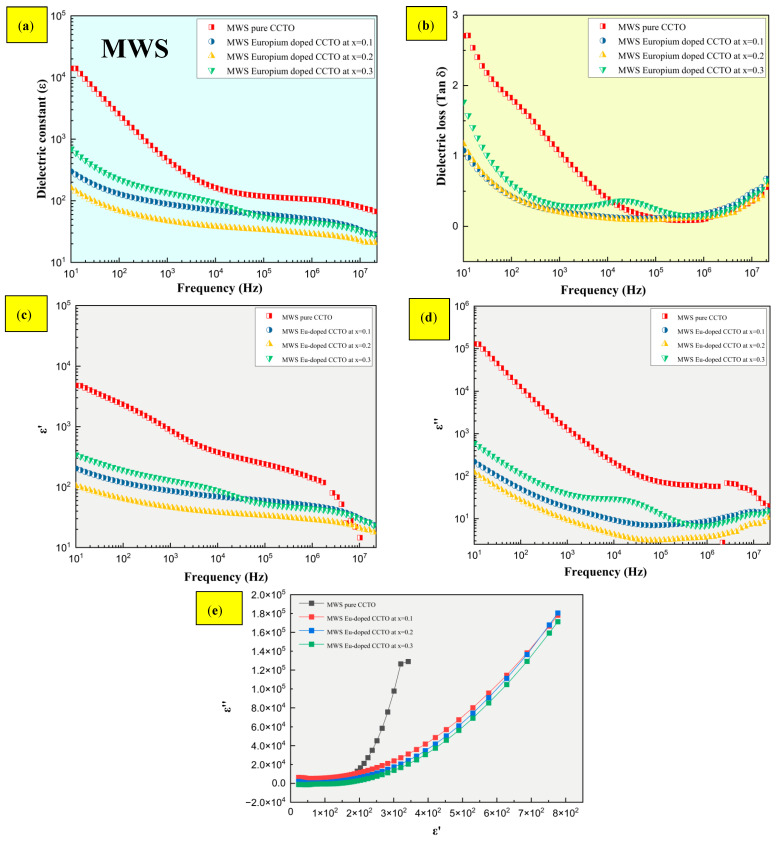
Dielectric properties of MWS europium-doped samples from x = 0.1 to 0.3 measured at room temperature. (**a**) ε/Frequency, (**b**) tan δ/frequency, (**c**,**d**) dielectric relaxation peaks, and (**e**) Cole-Cole plot.

Dielectric characteristics of standard sintered samples with effective doping of europium at x = 0.1 are shown in Figure 8a,b, where a high dielectric constant is seen (ε) > 10^2^ than pure CCTO without dopant. Significantly, the dielectric constant elevates at higher frequency from 10^4^ to 10^7^ Hz with a drop in dielectric loss. Loss is minimized even at higher frequencies, as seen by the fact that tan δ decreases for all three europium compositions at x = 0.1, 0.2, and 0.3. All conventional sintered europium-doped samples exhibited high ε > 10^4^ at 10 Hz as a function of frequency at x = 0.1 measured at room temperature. Similarly, the grain size, one of the material’s intrinsic features, was found to rise by more than a factor of 10^2^ as the europium content inflated from 0.2 to 0.3. In contrast, as shown in Figure 9a,b, the dielectric constant of MWS samples expanded with the increasing levels of Eu dopant at x > 0.1. The tan δ drastically drops at high frequency for all compositions of europium at x > 0.1. Subsequently, the dielectric relaxation peaks and Cole-Cole plot have been depicted in Figure 8c–e and Figure 9c–e for both conventional and microwave sintered samples. In Figure 8c–e and Figure 9c–e, the dielectric relaxation effect at medium (10^3^ Hz) and high (10^6^ Hz) frequencies have been suppressed by the inclusion of Eu^3+^ into the CCTO structure. It has been revealed that the interfacial polarization due to charge build-up causes the high dielectric constant to peak at low frequencies. When movement of Ti ions becomes arrested, the Debye relaxation behavior causes the dielectric constant to drop at high frequencies [36]. Cation valence state, oxygen vacancies [37], and the electrode contact point [38,39] have been associated with the low dielectric constant. This research work aims at promoting material with low dielectric loss at elevated frequency ranges. The more linear-like (less exponential-like) growth of *ε* in Cole-Cole plots was an indication of the less lossy character of MW samples compared to CS samples. One of the main goals of our study—to prepare CCTO-based samples which exhibit high and frequency-stable dielectric constant at simultaneously low losses—was demonstrated this way.

## 3. Materials and Methods

### 3.1. Material Preparation

Very small amounts of europium oxide dopant of different compositions at x = 0.1, 0.2, and 0.3 have been mixed with the CCTO powder with particle size of 400–800 nm (American Elements, Los Angeles, CA, USA, purity grade 99%). The precursor powders were amalgamated in a mortar and pestle for an hour to ensure proper blending of the dopant and ceramic powder. The powder combination was compressed into green compacts 15 mm in diameter and 5 mm in thickness using a hydraulic press (Technosearch Instruments, Model M-15, Mumbai, India) at a uniaxial pressure of 50 MPa. 

### 3.2. Sample Processing and Characterization

Muffle and microwave furnaces (VB CERAMIC CONSULTANTS, Chennai, India) were used to sinter the green pellets at 1100 °C for 12 h and 30 min, respectively. The microwave and traditional sintering heating rates were 50 and 5 °C min^−1^, respectively. Studies employing X-ray diffraction (XRD) were performed using the X-ray diffractometer (Bruker D8 Advance, Karlsruhe, Germany equipped with a 2.2 kW Cu anode and Ni filter over the 2ϴ range of 20–80°, with a step size of 0.05. Field emission-scanning electron microscope (FE-SEM) (Thermo Fisher FEI QUANTA 250 FEG) at 20 keV was used for microstructure investigation and energy dispersive spectroscopy (EDS) analysis of europium-doped CCTO samples. Samples were sputter coated with a thin film of noble metals to offer conductivity before microscopic analysis. Moreover, the samples were thermally etched at 950 °C for 15 min in a standard furnace to easily distinguish the grain and grain boundaries. For the purpose of determining the bonding energies of valence electrons and their function in IBLC, X-ray photoelectron spectroscopy (XPS; ULVAC-PHI, Inc; Model: PHI5000 Version Probe 94 III) was employed in conjunction with an Al monochromatic radiation source at 280 eV. Broadband dielectric spectrometer (BDS) measurements included dielectric constant (ε), tan δ, and relaxation peaks (Concept 80, Novocontrol Technologies, Montabaur, Germany). A silver adhesive paste of sheet resistance 0.25/square at 0.001 mm in thickness (Thermo Fisher Scientific India Pvt Ltd., Mumbai, India) was applied to both ends of the pellets, and then heat treated at 150 °C for 1 h to generate electrodes, which facilitated the dielectric measurement.

## 4. Conclusions

The following inferences were driven from this research work. The difference in grain size between CS and MW samples was dramatic, whereas the MW sintering led to finer grains. However, this difference was not reflected in a similarly strong way in the difference of dielectric properties.

- The absence of Cu phase segregation at the grain boundaries was confirmed by XRD plots and elemental mapping. In addition, the evolution of microstructure fostered by the inclusion of europium in the CCTO structure led to smaller grains in the CS samples at x > 0.2 and elevated for MWS samples at x > 0.1. In the case of the absence of grain boundaries with different electro-chemical characters, the phenomena taking place at atomic lattice level are responsible for the dielectric behavior of samples.
- The charge compensation effect, which is facilitated by reduction processes, have been confirmed by XPS analysis for Ti^4+^ and Cu^2+^ that directly impacts the dielectric properties.
- The dielectric constant ε was greater than 10^4^ and 10^2^ for CS and MWS samples, with stable low tan δ at high frequencies ranging from 10^5^ to 10^7^ Hz. Although the presence of Eu^3+^ induces low tan δ at high frequencies, boosting the dielectric constant is dependent on a number of factors, which include the presence of oxygen vacancies, and the type and area of contact of the electrodes with the sample.

## Data Availability

Data will be subjected to availability on request to the corresponding author.
